# Supplementary figures and images for: Empowering beginners in bioinformatics with ChatGPT
Source: Quant Biol. 2023 Jun 1;11(2):105–8. doi: 10.15302/J-QB-023-0327 (PMC10299548; doi:10.15302/J-QB-023-0327)

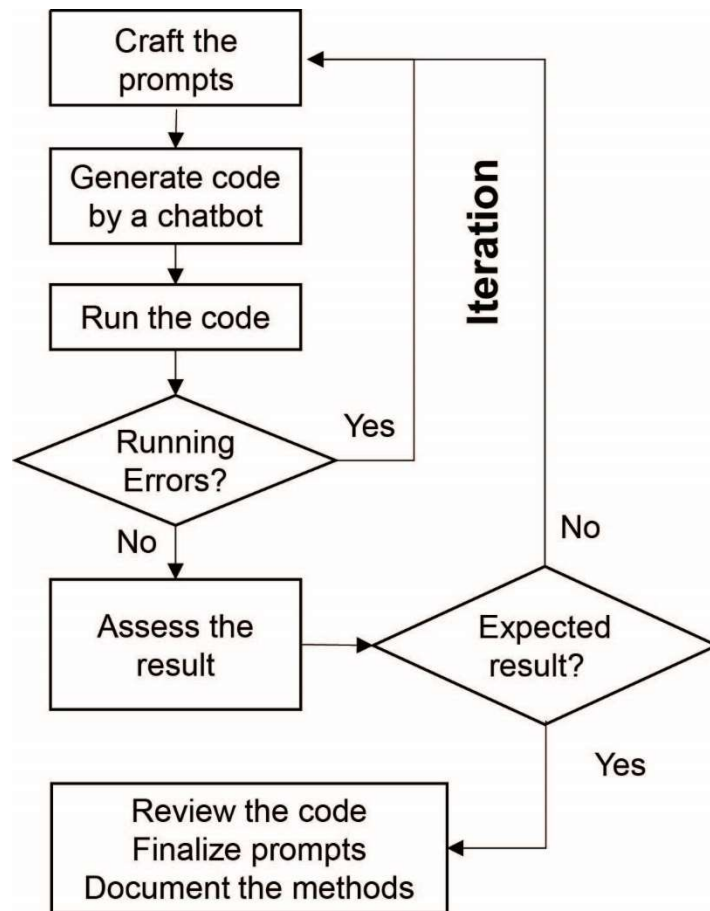

Figure S1: The OPTIMAL model for LLM chatbot-assisted scientific data analysis

Supplement: Supplementary file 1 — Supplementary Information [file QUB2-11-105-s006.pdf]
