# Supplementary material for: Empowering beginners in bioinformatics with ChatGPT
Source: Quant Biol. 2023 Jun 1;11(2):105–8. doi: 10.15302/J-QB-023-0327 (PMC10299548; doi:10.15302/J-QB-023-0327)
Supplement: Supplementary file 4 — Supplementary Information [file QUB2-11-105-s007.pdf]

**Table S2: OPTIMAL CASE STUDY: Phylogeny inference based on TP53**
**Description:**<br>Test how ChatGPT can help students build a phylogenetic tree starting with aligned TP53 gene coding sequences from 9 species.<br><br>**Coding language(s):**<br>R
**PREREQUISITE KNOWLEDGE**
To complete this task, students need to have fundamental knowledge of molecular evolution and phylogenetic analysis, understanding of R syntax, and basic debugging skills.
Prompts to d*efine chatbot's behaviors*
Act as an experienced bioinformatician proficient in R, you will write code with number of lines as minimal as possible. Rest the thread if asked to. Reply "YES" if understand.
**ITERATION 0**
**Prompts**
You have a multiple alignment file named as tp53.clustal in ClustalW format. Please write R code that can load the file, calculate evolutionary distance, build a NJ tree, and visualize the phylogeny.
**Code**
Load the required packages<br>library(ape)<br><br># Read in the alignment file<br>aln <- read.alignment("tp53.clustal", format="clustal")<br><br># Calculate the evolutionary distance<br>dist <- dist.dna(aln)<br><br># Build the NJ tree<br>tree <- nj(dist)<br><br># Plot the phylogeny<br>plot(tree)
**Result**
Failed with an error message.
**ITERATION 1**
**Prompts**
I got an error message complaining "could not find function "read.alignment". Please fix it.
**Code**
Load the required packages<br>library(seqinr)<br>library(ape)<br><br># Read in the alignment file<br>aln <- read.alignment("tp53.clustal", format="clustal")

```
# Calculate the evolutionary distance using the Jukes-Cantor distance
dist <- dist.dna(aln, model="JC")

# Build the NJ tree
tree <- nj(dist)

# Plot the phylogeny
plot(tree)
```

Result

Failed with an error message.

ITERATION 2

Prompts

I got a warning message " In dist.dna(aln) : NAs introduced by coercion". Please fix it.

Code

```
# Load the required packages
library(seqinr)
library(ape)

# Read in the alignment file
aln <- read.alignment("tp53.clustal", format="clustal")

# Calculate the evolutionary distance using the Jukes-Cantor distance
dist <- dist.dna(aln, model="JC")

# Build the NJ tree
tree <- nj(dist)

# Plot the phylogeny
plot(tree)
```

Result

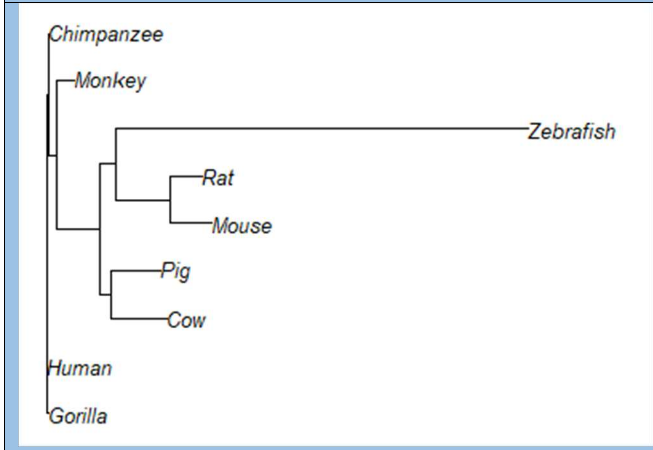

The code displays an unroot tree. Manual editing of the code and instruct ChatGPT to generate a rooted tree.

ITERATION 3

Prompts
I wrote an R program to read a multiple alignment file named as tp53.clustal in ClustalW format, calculate evolutionary distance, build a NJ tree, and visualize the phylogeny. But I want to root the tree with the Zebrafish sequence as the outgroup. Can you help me revise the R code? Below is my R code.<br># Load the required packages<br>library(seqinr)<br>library(ape)<br><br># Read in the alignment file<br>aln <- read.alignment("tp53.clustal", format="clustal")<br><br># Calculate the evolutionary distance<br>dist <- dist.dna(as.DNAbin(aln))<br><br># Build the NJ tree<br>tree <- nj(dist)<br><br># Plot the phylogeny<br>plot(tree)
**Codes**
Load the required packages<br>library(seqinr)<br>library(ape)<br><br># Read in the alignment file<br>aln <- read.alignment("tp53.clustal", format="clustal")<br><br># Identify the index of the Zebrafish sequence in the alignment<br>zebrafish_idx <- which(aln$species == "Danio_rerio")<br><br># Calculate the evolutionary distance<br>dist <- dist.dna(as.DNAbin(aln))<br><br># Build the NJ tree<br>tree <- nj(dist, outgroup = zebrafish_idx)<br><br># Plot the phylogeny<br>plot(tree)
**Results**
Failed with an error message.
**ITERATION 4**
**Prompts**
I got an error message complaining " Error in nj(dist, outgroup = zebrafish_idx) unused argument (outgroup = zebrafish_idx)". Please fix it.
**Codes**
Load the required packages<br>library(seqinr)<br>library(ape)

```
# Read in the alignment file
aln <- read.alignment("tp53.clustal", format="clustal")

# Identify the index of the Zebrafish sequence in the alignment
zebrafish_idx <- which(aln$species == "Danio_rerio")

# Calculate the evolutionary distance
dist <- dist.dna(as.DNAbin(aln))

# Build the NJ tree
tree <- nj(dist)

# Root the NJ tree with the Zebrafish sequence as the outgroup
tree_rooted <- root(tree, outgroup = zebrafish_idx)

# Plot the phylogeny
plot(tree_rooted)
```

Results

Failed with an error message.

ITERATION 5

Prompts

I got an error message complaining "Error in if (newroot == ROOT) { : argument is of length zero". Please fix it.

Codes

```
# Load the required packages
library(seqinr)
library(ape)

# Read in the alignment file
aln <- read.alignment("tp53.clustal", format="clustal")

# Identify the index of the Zebrafish sequence in the alignment
zebrafish_idx <- which(aln$species == "Danio_rerio")

# Calculate the evolutionary distance
dist <- dist.dna(as.DNAbin(aln))

# Build the NJ tree
tree <- nj(dist)

# Root the NJ tree with the Zebrafish sequence as the outgroup
tree_rooted <- root(tree, outgroup = zebrafish_idx)

# Plot the phylogeny
plot(tree_rooted)
```

Results

Failed with an error message.

Manual editing of the code.
The code displays a correctly rooted tree.

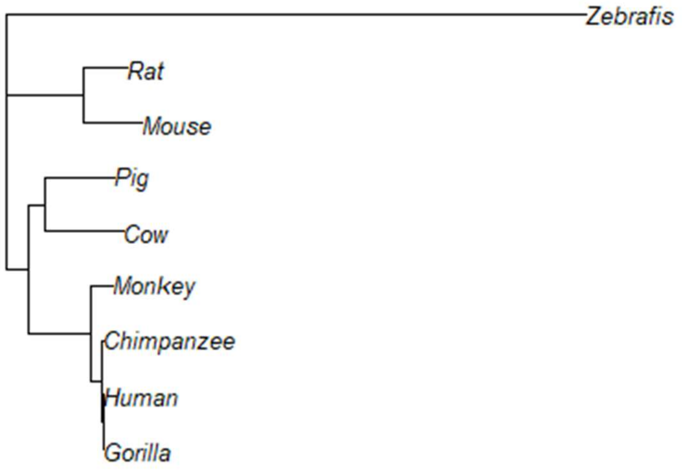

WRAPPING UP

Final codes with explanation to review:

Prompt: I created the following R code. Please add inline comments.

```
library(seqinr)
library(ape)
aln <- read.alignment("tp53.clustal", format = "clustal")
dist_matrix <- dist.dna(as.DNAbin(aln))
zebrafish_index <- which(attr(dist_matrix, 'Labels') == "Zebrafish")
tree <- nj(dist_matrix)
tree_rooted <- root(tree, zebrafish_index)
plot(tree_rooted, cex = 0.8)
```

```
# Load the required packages
library(seqinr)
library(ape)

# Read in the alignment file
aln <- read.alignment("tp53.clustal", format = "clustal")

# Calculate the evolutionary distance matrix
dist_matrix <- dist.dna(as.DNAbin(aln))

# Identify the index of the Zebrafish sequence in the distance matrix labels
zebrafish_index <- which(attr(dist_matrix, 'Labels') == "Zebrafish")

# Build the NJ tree
tree <- nj(dist_matrix)

# Root the NJ tree with the Zebrafish sequence
tree_rooted <- root(tree, zebrafish_index)

# Plot the phylogeny with modified label size
plot(tree_rooted, cex = 0.8)
```

Summarize the question, methods, and results

In this session, we aim to assess how ChatGPT can help beginners to build a phylogenetic tree starting with a set of aligned sequences of the TP53 gene from 9 species. We started with a prompt that specified the analysis steps and asked ChatGPT to generate R code. We then copied the code and

tested it in R. If the execution generated an error or a warning, we presented the message to ChatGPT and requested it to fix the problem. If ChatGPT couldn't fix the problem after two iterations, we manually edited the code.
**Additional Comments**
1. ChatGPT was able to solute easy problems such as calling a function in a library but not loading the library beforehand.<br>2. ChatGPT struggled to resolve more complicated problems, such as identifying and converting to correct data types, and setting advanced parameters in function calls.<br>3. ChatGPT sometimes made up functions that did not exist.
