# Supplementary material for: Empowering beginners in bioinformatics with ChatGPT
Source: Quant Biol. 2023 Jun 1;11(2):105–8. doi: 10.15302/J-QB-023-0327 (PMC10299548; doi:10.15302/J-QB-023-0327)
Supplement: Supplementary file 6 — Supplementary Information [file QUB2-11-105-s002.pdf]

**Table S4: OPTIMAL CASE STUDY: On Relationship Between Household Income and High School Graduation Rates**
**Description**<br><br>This case study aims to explore the relationship between median household income and high school graduation rates in Texas counties from 2020 to 2022, using rigorous regressions and intuitive graphs.<br><br>Regression analysis is a long-established and widely used quantitative data analysis technique in the social sciences. It is a compelling statistical procedure that enables researchers to describe, predict, and estimate the relationships between one or more independent variables and a dependent variable and draw plausible conclusions. Moreover, catterplots are one of the most common types of graphs that depicts the relationships between two variables. A nice scatterplot conveys the correlation, the spread, and outliers at the same time.<br><br>The case study demonstrates beginners in data analysis with cross-section data by estimating the Ordinary Least Square (OLS) regression, with panel data (longitudinal data) that contains observations about different cross sections across time by conducting panel regression, and with a nice two-way scatterplot.<br><br>In the multivariate regressions, the estimated coefficients for median household income are positive but statistically insignificant. When looking solely at the two-way scatterplot, median household income is positively associated with high school graduation rates.<br><br>**Coding language(s)**<br><br>Stata
**PREREQUISITE KNOWLEDGE**
To complete the task, students should have a foundational understanding of econometrics, specifically in the areas of correlational analysis between two variables, pooled OLS regressions, and panel fixed effect regressions.
Prompts to d*efine chatbot's behaviors*
Act as an experienced economist proficient in Stata, you will write code with a number of lines as minimal as possible.<br>The input file(s) description will be [like this].<br>Reply "Input received" when receiving text with [] and explain.<br>Details of data analysis will be {like this}.<br>Reply "Analysis request received" when receiving text within {}, explain the text, followed by generating the code.<br>Reply "Yes" if understood.
**INPUT FILE**

Prompts

[I have an Excel file: County Level Data.xls. I have variable names in the first row of my worksheet. The variable names are CountyID, year, HighSchoolGraduationRate, MedianHouseholdIncome, IncomeInequalityRatio, and SingleParentHouseholdRatio.]

Code

N/A

Results

N/A

Assessments

N/A

REQUEST 1

Prompts

{Run an OLS regression of HighSchoolGraduationRate on MedianHouseholdIncome, IncomeInequalityRatio, and SingleParentHouseholdRatio.}

Code

```
import excel "County Level Data.xls", sheet("Sheet1") firstrow clear

reg HighSchoolGraduationRate MedianHouseholdIncome IncomeInequalityRatio
SingleParentHouseholdRatio
```

Result

```
. import excel "County Level Data.xls", sheet("Sheet1") firstrow clear
(7 vars, 633 obs)

. reg HighSchoolGraduationRate MedianHouseholdIncome IncomeInequalityRatio SingleParentHouseholdRatio

      Source |       SS           df       MS      Number of obs   =       530
-------------+----------------------------------   F(3, 526)       =     10.45
       Model |  571.028314         3  190.342771   Prob > F        =    0.0000
    Residual |  9578.98846       526  18.2110047   R-squared       =    0.0563
-------------+----------------------------------   Adj R-squared   =    0.0509
       Total |  10150.0168       529  19.1871773   Root MSE        =    4.2674

------------------------------------------------------------------------------------------
  HighSchoolGraduationRate |      Coef.   Std. Err.      t    P>|t|     [95% Conf. Interval]
---------------------------+--------------------------------------------------------------
     MedianHouseholdIncome |  -7.20e-06   .0000173    -0.42   0.676    -.0000411    .0000267
     IncomeInequalityRatio |  -.6429799   .2843825    -2.26   0.024    -1.201645    -.084315
SingleParentHouseholdRatio |  -.0968013   .0265715    -3.64   0.000    -.1490007    -.044602
                     _cons |   99.21074   2.005443    49.47   0.000     95.27108    103.1504
------------------------------------------------------------------------------------------
```

Assessment

The code runs successfully.
The Excel file is successfully imported into Stata in numerical format.
The code to conduct the OLS regression analysis is correct.

REQUEST 2

Prompts

{How about run a panel regression with year fixed effects?}

Code

```
import excel "County Level Data.xls", sheet("Sheet1") firstrow clear

xtset CountyID year
xtreg HighSchoolGraduationRate MedianHouseholdIncome IncomeInequalityRatio
SingleParentHouseholdRatio i.year, fe
```

Result

```
. xtset CountyID year
       panel variable:  CountyID (unbalanced)
        time variable:  year, 2020 to 2022, but with gaps
                delta:  1 unit

. xtreg HighSchoolGraduationRate MedianHouseholdIncome IncomeInequalityRatio SingleParentHouseholdRatio i.year, fe

Fixed-effects (within) regression               Number of obs     =        530
Group variable: CountyID                        Number of groups  =        230

R-sq:                                           Obs per group:
     within  = 0.0079                                         min =          1
     between = 0.0533                                         avg =        2.3
     overall = 0.0461                                         max =          3

                                                F(5,295)          =       0.47
corr(u_i, Xb)  = 0.0336                         Prob > F          =     0.7994

----------------------------------------------------------------------------------------------
  HighSchoolGraduationRate |      Coef.   Std. Err.      t    P>|t|     [95% Conf. Interval]
---------------------------+------------------------------------------------------------------
     MedianHouseholdIncome |   .0000462     .00005     0.93   0.356    -.0000521    .0001446
     IncomeInequalityRatio |   .2019583   .4369435     0.46   0.644    -.6579633     1.06188
SingleParentHouseholdRatio |  -.0451537   .0420321    -1.07   0.284    -.1278744     .037567
                           |
                      year |
                     2021  |  -.6526546    .462255    -1.41   0.159     -1.56239    .2570808
                     2022  |  -.7510443   .5287916    -1.42   0.157    -1.791726    .2896377
                           |
                     _cons |    91.3979   3.635723    25.14   0.000     84.24266    98.55315
---------------------------+------------------------------------------------------------------
                   sigma_u |   3.996722
                   sigma_e |  2.3946805
                       rho |  .73583838   (fraction of variance due to u_i)
----------------------------------------------------------------------------------------------
F test that all u_i=0: F(229, 295) = 5.73                    Prob > F = 0.0000
```

Assessment

The code run successfully. The panel regression analysis is completed.

REQUEST 3 (ITERATION 0)

Prompts

{Show the relationship between HighSchoolGraduationRate and MedianHouseholdIncome in a figure}

Code

```
import excel "County Level Data.xls", sheet("Sheet1") firstrow clear

tw scatter HighSchoolGraduationRate MedianHouseholdIncome, xtitle("Median Household Income") ytitle("High School Graduation Rate") graphregion(margin(large)) name(scatterplot, replace)
```

Result

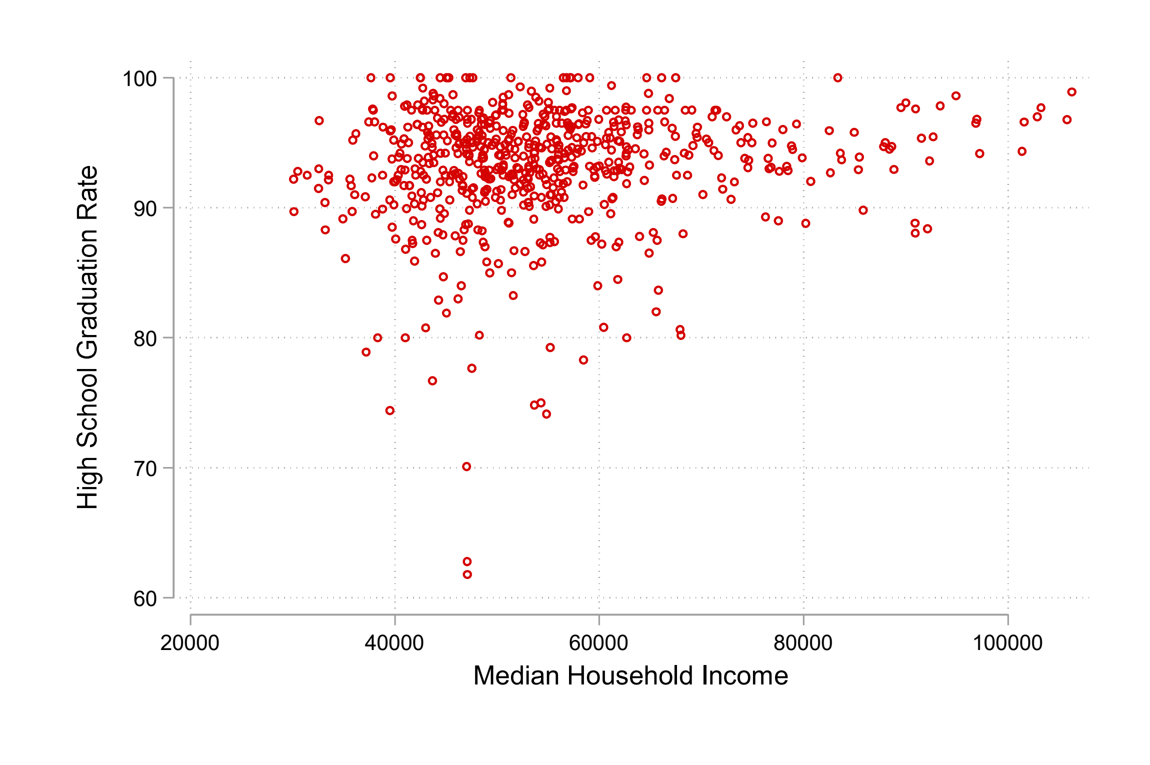

Assessment

The code run successfully.
The two-way scatterplots display the joint distribution of the HighSchoolGraduationRate and MedianHouseholdIncome variables.

REQUEST 3 (ITERATION 1)

Prompt

{Add a title "High School Graduation Rate and Median House hold Income", a subtitle "County Level Evidence from Texas, 2020-2022", and a regression line and in the figure.}

Code

```
import excel "County Level Data.xls", sheet("Sheet1") firstrow clear
```

```
regress HighSchoolGraduationRate MedianHouseholdIncome

twoway (scatter HighSchoolGraduationRate MedianHouseholdIncome, xtitle("Median
Household Income") ytitle("High School Graduation Rate") graphregion(margin(large))
name(scatterplot, replace)) (lfit HighSchoolGraduationRate MedianHouseholdIncome),
title("High School Graduation Rate and Median Household Income") subtitle("County Level
Evidence from Texas, 2020-2022")
```

Result

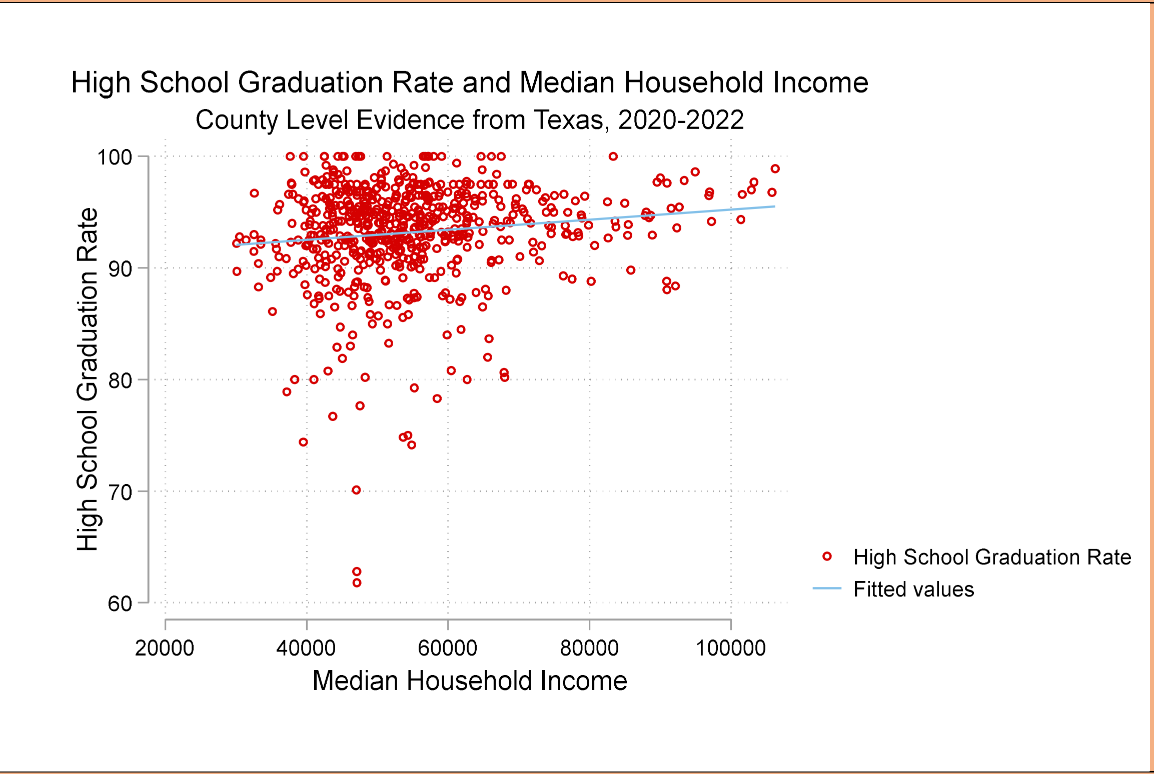

Assessment

The code for the OLS regression is unnecessary.

All other codes are good.

WRAPPING UP

Review the final code

Review the comments added by chatGPT and identify key steps, as represented by functions and parameters, that are missing in the initial prompts.

Prompts

{I need to insert line-by-line comments to the below code which works well to address the needs for the data analysis task. Wait for my code.}

```
import excel "County Level Data.xls", sheet("Sheet1") firstrow clear
reg HighSchoolGraduationRate MedianHouseholdIncome IncomeInequalityRatio
SingleParentHouseholdRatio
xtset CountyID year
xtreg HighSchoolGraduationRate MedianHouseholdIncome IncomeInequalityRatio
SingleParentHouseholdRatio i.year, fe
twoway (scatter HighSchoolGraduationRate MedianHouseholdIncome, xtitle("Median
Household Income") ytitle("High School Graduation Rate") graphregion(margin(large))
name(scatterplot, replace)) (lfit HighSchoolGraduationRate MedianHouseholdIncome),
title("High School Graduation Rate and Median Household Income") subtitle("County Level
Evidence from Texas, 2020-2022")
```

Code with explanations

```
// Import the data from the "County Level Data.xls" file
// with the variable names in the first row of the worksheet
import excel "County Level Data.xls", sheet("Sheet1") firstrow clear

// Run a simple linear regression of HighSchoolGraduationRate on MedianHouseholdIncome,
// IncomeInequalityRatio, and SingleParentHouseholdRatio
reg HighSchoolGraduationRate MedianHouseholdIncome IncomeInequalityRatio
SingleParentHouseholdRatio

// Set the data as a panel with CountyID as the panel variable and year as the time variable
xtset CountyID year

// Run a fixed effects panel regression of HighSchoolGraduationRate on
MedianHouseholdIncome,
// IncomeInequalityRatio, and SingleParentHouseholdRatio, with year fixed effects
xtreg HighSchoolGraduationRate MedianHouseholdIncome IncomeInequalityRatio
SingleParentHouseholdRatio i.year, fe

// Create a scatter plot of HighSchoolGraduationRate against MedianHouseholdIncome
// with x and y axis titles and a large graph region, and save it as "scatterplot"
// Then, overlay a linear regression line on top of the scatter plot and add a title
// and subtitle to the graph
twoway (scatter HighSchoolGraduationRate MedianHouseholdIncome, xtitle("Median
Household Income") ytitle("High School Graduation Rate") graphregion(margin(large))
name(scatterplot, replace)) (lfit HighSchoolGraduationRate MedianHouseholdIncome),
title("High School Graduation Rate and Median Household Income") subtitle("County Level
Evidence from Texas, 2020-2022")
```

SUMMARY

Aims

The goal of this session is to input data in the Excel format in the statistical software and then run regression analysis and create an intutitive graph.

Methods

By providing appropriate prompts to ChatGPT, the case study demonstrates how beginners could obtain code from ChatGPT to run OLS regression for cross-section data and panel regression with time fixed effect for longitudinal data, along with data scatter plots.

Results and Discussions

Regression analysis is probably the oldest and most widely adopted quantitative data analysis technique in the social sciences. With the statistical method used to evaluate the relationship between the dependent variable and independent variables, beginners in data analysis can understand the significance of their data points and use analytical techniques to make better decisions.

The pattern of dots on a scatterplot, along with a fitted line for a simple regression model, is a frequently used and effective way to illustrate the relationship or correlation between two continuous variables.

The codes obtained from ChatGPT are satisfactory. Besides providing the codes, ChatGPT also gives a detailed explanation. The process dramatically helps beginners learn how to interact with ChatGPT to resolve their demands, such as inputting data and conducting analysis, in a short time.

Additional Comments

Students need to know the subject matter to craft the prompts and ask pertinent questions. Without clear instructions on the object of the analysis, ChatGPT will have difficulty providing the correct code.
